# Supplementary material for: A genetic circuit on a single DNA molecule as an autonomous dissipative nanodevice
Source: Nat Commun. 2024 Jan 29;15:883. doi: 10.1038/s41467-024-45186-2 (PMC10825189; doi:10.1038/s41467-024-45186-2)
Supplement: Supplementary file 1 — Supplementary Information [file 41467_2024_45186_MOESM1_ESM.docx]

**Supplementary Information for**

**A genetic circuit on a single DNA molecule as an autonomous dissipative nanodevice**

Ferdinand Greiss^1,*^, Nicolas Lardon^2^, Leonie Schütz^3^, Yoav Barak^4^, Shirley Shulman Daube^1^, Elmar Weinhold^3^, Vincent Noireaux^5^, Roy Bar-Ziv^1,*^

^1^Department of Chemical and Biological Physics, Weizmann Institute of Science, Rehovot, 7610001, Israel

^2^Department of Chemical Biology, Max Planck Institute for Medical Research, 69120 Heidelberg, Germany

^3^Institute of Organic Chemistry, RWTH Aachen University, 52056 Aachen, Germany

^4^Department of Chemical Research Support, Weizmann Institute of Science, Rehovot, 7610001, Israel

^5^School of Physics and Astronomy, University of Minnesota, Minneapolis, MN, 55455, USA

*Corresponding authors: F.G. (ferdinand.greiss@gmail.com), R.B.Z (roy.bar-ziv@weizmann.ac.il)


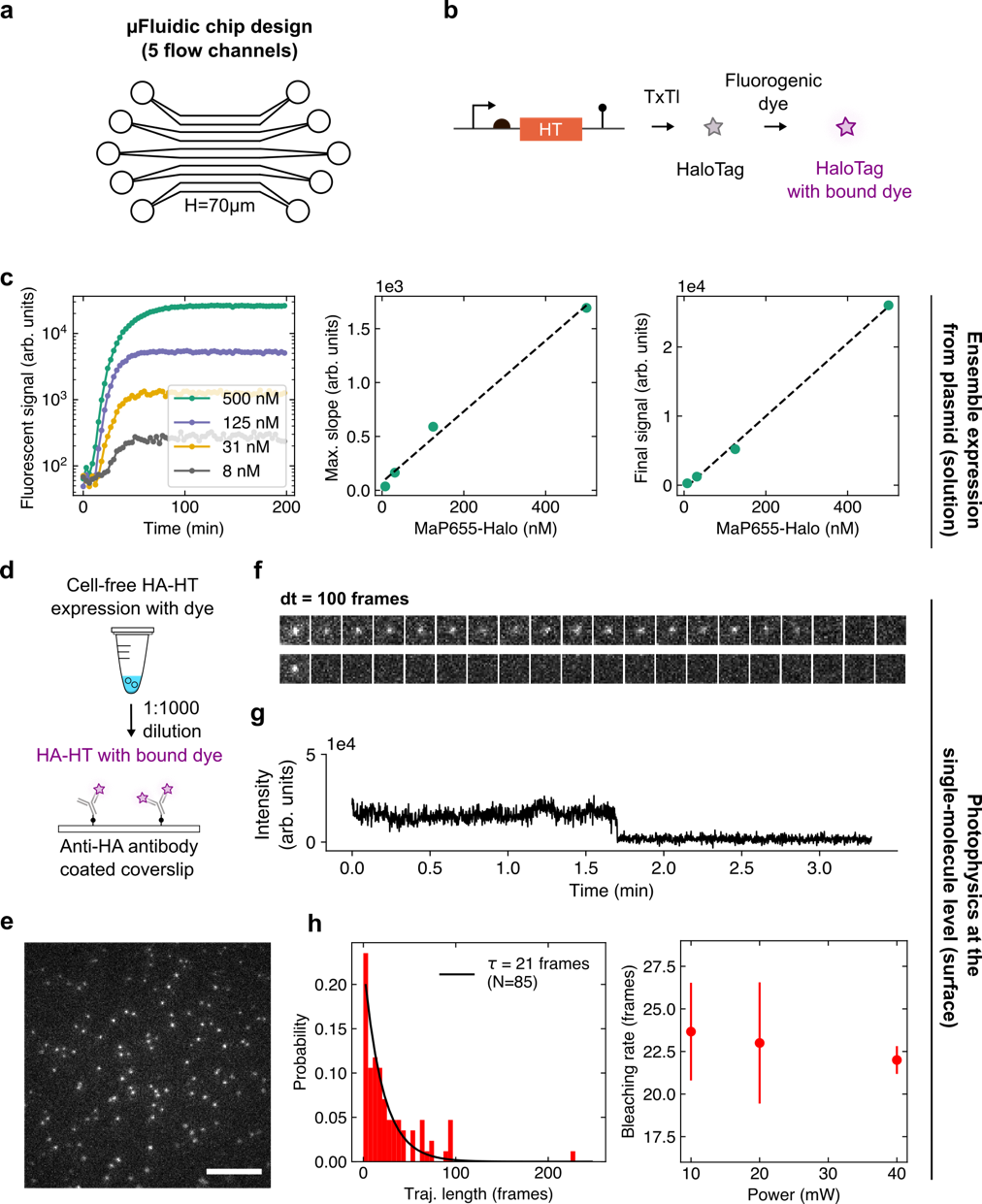


**Supplementary Figure 1: Cell-free expression and photo-physics of MaP655-Halo bound to HT proteins. a)** Microfluidic chip design with 5 parallel flow channels (L~6000 µm, W~600 µm). **b)** Gene expression from the *ht* cassette produces HT proteins that can rapidly bind the fluorogenic dye MaP655-Halo. The fluorogenic dye reacts with nascent HT proteins and increases its fluorescent signal by ~1,000-fold. **c)** Cell-free expression dynamics in bulk with various concentrations of MaP655-Halo. As expected, the maximal slope and final fluorescence signal of the expression curves (as shown in the leftmost panel) increased linearly with the concentration of MaP655-Halo. **d)** To measure the photo-stability of bound MaP655-Halo, HA-tagged HT was expressed in solution with 50 nM of fluorogenic dye. The DNA was expressed for ~1 h to produce enough HT to bind most free fluorogenic dye. The mix was diluted by 1,000-fold with PBS and flushed on an anti-HA antibody coated coverslip. **e)** Fluorescence microscopy image of immobilized HT protein bound to MaP655-Halo. Scale bar, 10 µm. **f)** Two exemplary HT traces with characteristic single-molecule bleaching steps. **g)** The intensity trace of a single HT spot over time. **h)** Bleaching rate of the dye bound to HT and fit to mono-exponential decay (black line) at an excitation power of 15 W cm^-2^ (equals an input power of 10 mW). The bleaching rate for different laser input powers. The average and error bars as SD of fitted rates from mono-exponential decay curves as indicated in left graph (n=3 independent experiments).


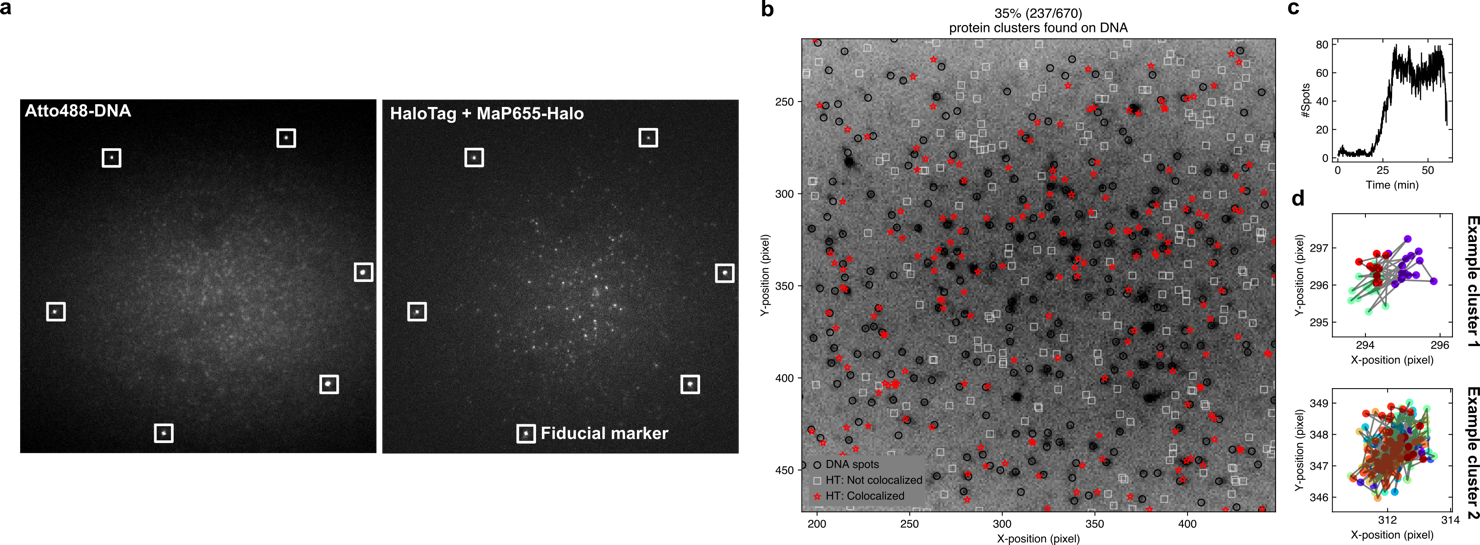


**Supplementary Figure 2: The detection and analysis of protein synthesis spots on the surface of a flow channel. a)** Fluorescent image of DNA molecules and HT protein synthesis spots with fiducial markers (white boxes). **b)** Fluorescence microscopy image of DNA distribution at the beginning of an experiment. The identified DNA spots are indicated by black circles. Colocalized and non-colocalized HT spots are indicated by red stars and white squares, respectively. **c)** The number of detected HT spots over time. The start of expression is marked by an increase of protein synthesis spots on the surface. After tens of minutes, a balance between protein production and removal is reached, maintaining a steady-state protein level on the coverslip surface. **d)** Two examples for identified protein synthesis spots (cluster). The colors indicate protein synthesis interspersed by periods without protein synthesis near the same location (one color corresponds to one continuous period of protein synthesis). The individual trajectories are clustered together to define a protein synthesis spot. The average positions for each cluster are used to compute the protein synthesis intensity traces.


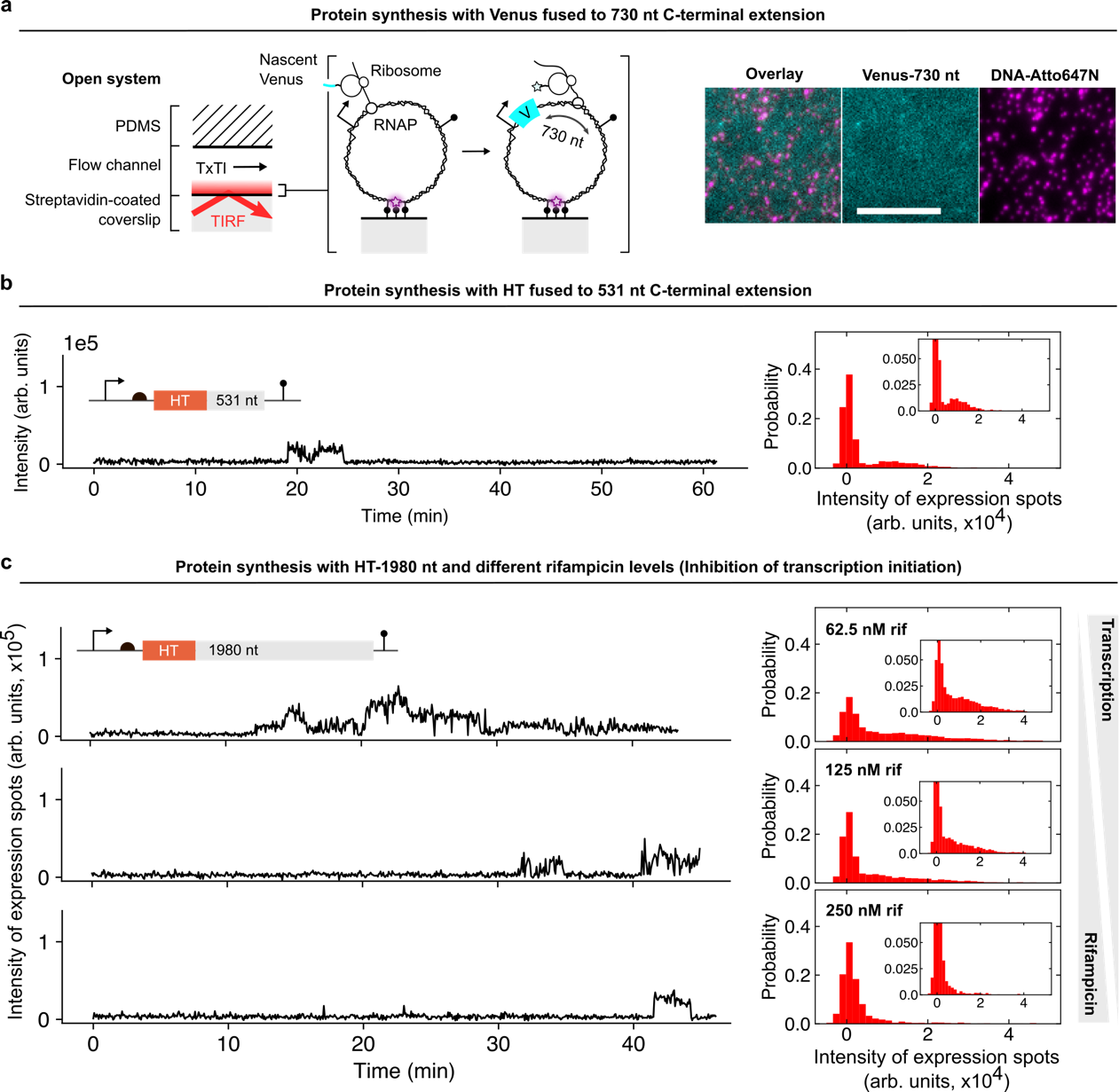


**Supplementary Figure 3: Single-molecule gene expression from DNA molecules encoding Venus, HT fused to a 531 nt long C-terminal extension, and HT fused to 1,980 nt long C-terminal extension with various rifampicin concentrations. a)** Single-molecule gene expression experiment with Venus fused to a 730 nt long C-terminal fusion encoded on DNA molecules with dC-Atto647N fluorescent labels. Scale bar, 10 µm. **b)** Exemplary intensity trace from a single DNA molecule and intensity probability distribution from an ensemble of DNA molecules encoding the *ht* gene fused to a 531 nt-long C-terminal extension. **c)** Exemplary intensity traces for protein synthesis spots (left column) with the 1,980 nt long C-terminal fused to HT at three different rifampicin concentrations as indicated in the intensity probability distributions assembled from an ensemble of DNA molecules (right column).


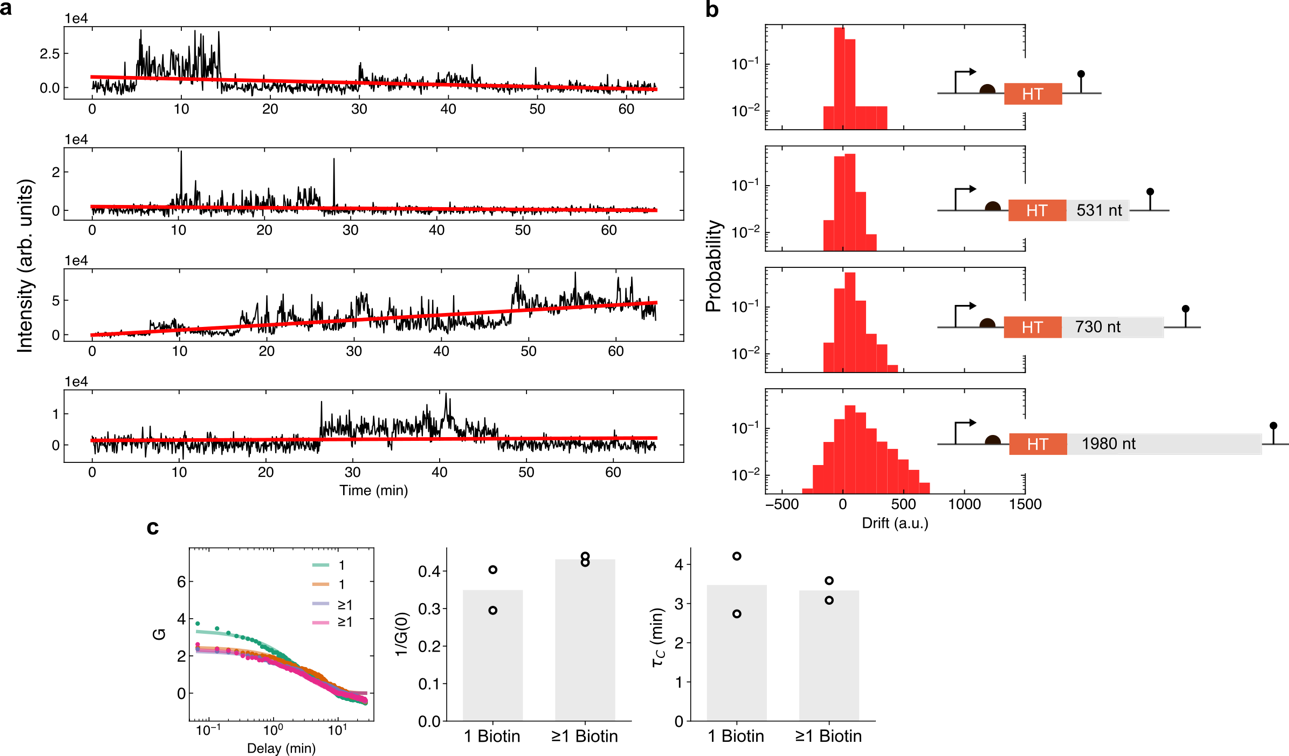


**Supplementary Figure 4: Accumulation of protein signal with different C-terminal extension fused to the HT protein. a)** Exemplary intensity traces for protein synthesis spots with the 2,021 nt long C-terminal fusion and their linear fits (red line). **b)** The slopes of all gene expression spot extracted from the linear fits, plotted as histograms for the four different gene constructs as schematically indicated. **c)** The ACF, inverse amplitude G(0)^-1^, and correlation time τ_C_ (extracted from mono-exponential fits to ACFs) as described in the methods and main text for the 2,021 nt long C-terminal fusion length and different numbers of biotins for surface immobilization. Experiments were replicated from left to right for n=2,2 independent experiments (small circles) to obtain mean values (bars).
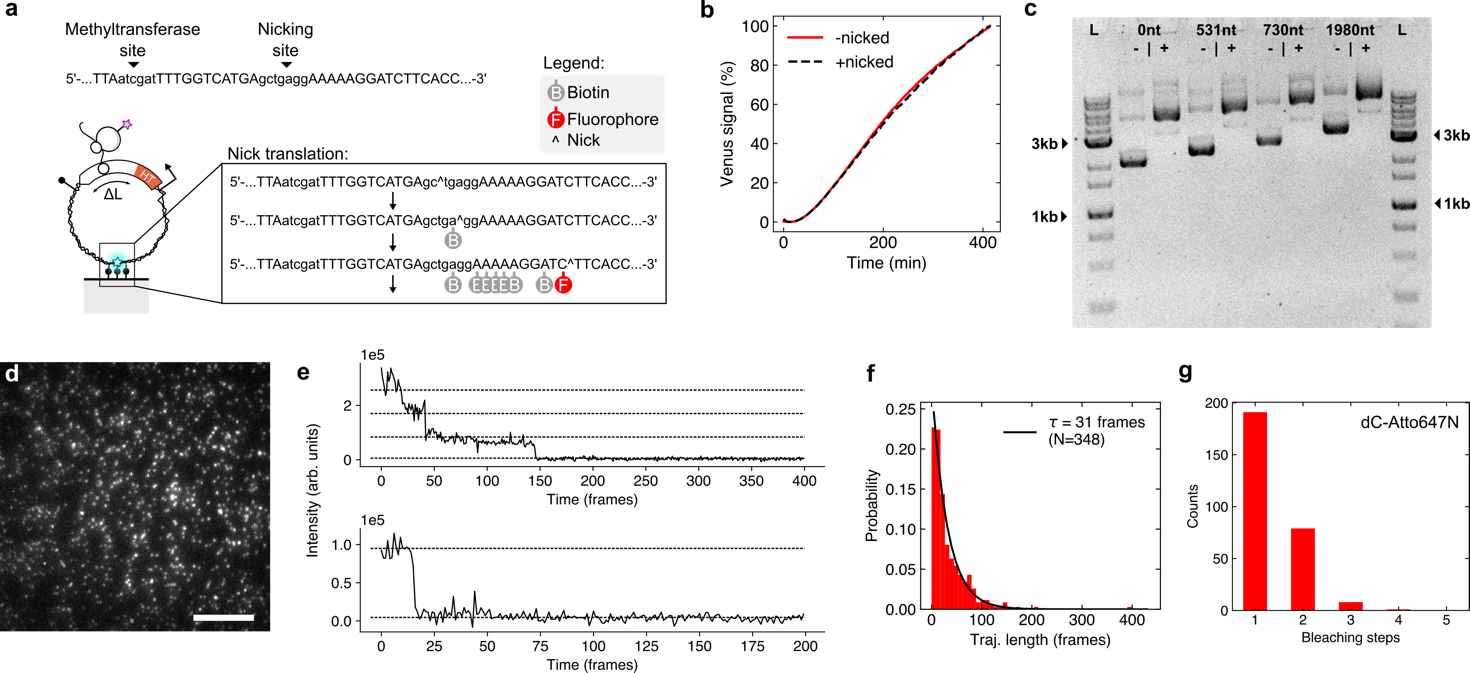


**Supplementary Figure 5: DNA labeling density. a)** Schematic of DNA molecule for surface-immobilization with zoom on sequence containing the two unique sites for the methyltransferase enzyme (M.BseCI) for biotinylating DNA molecules with a single biotin and nicking enzyme (Nb.BbvCI) for modifications with multiple biotins and fluorescent labels (Box: Schematic steps during nick translation using dA-Biotin and dC-Atto647N nucleotides for modification, as in panel d-g). **b)** Cell-free gene expression in solution from DNA with and without nick. **c)** Agarose gel with DNA encoding the *ht* gene with the four C-terminal fusion tags. The lanes correspond to DNA without (-) and with (+) nicking enzyme after nick translation. The DNA was labeled with Ethidium Bromide and run with a DNA ladder (L). As quality control, such band shifts were observed for every DNA preparation. **d)** Image of Atto647N-labeled DNA molecules immobilized on the surface during gene expression. DNA immobilization was replicated multiple times with similar results (n=4). Scale bar, 10 µm. **e)** Typical bleaching trajectories of single DNA. As expected for single dyes, the intensity during imaging exhibited discrete bleaching steps (indicated with dashed lines). **f)** The trajectories were extracted and their time until completely bleached was plotted as histogram. The lifetimes were fitted to a mono-exponential decay and gave a typical fluorescent lifetime (=1/bleaching rate) of 31 frames with the excitation parameters that was used in all single-molecule experiments. **g)** Estimated number of fluorophores (by counting bleaching steps) on single DNA molecules.


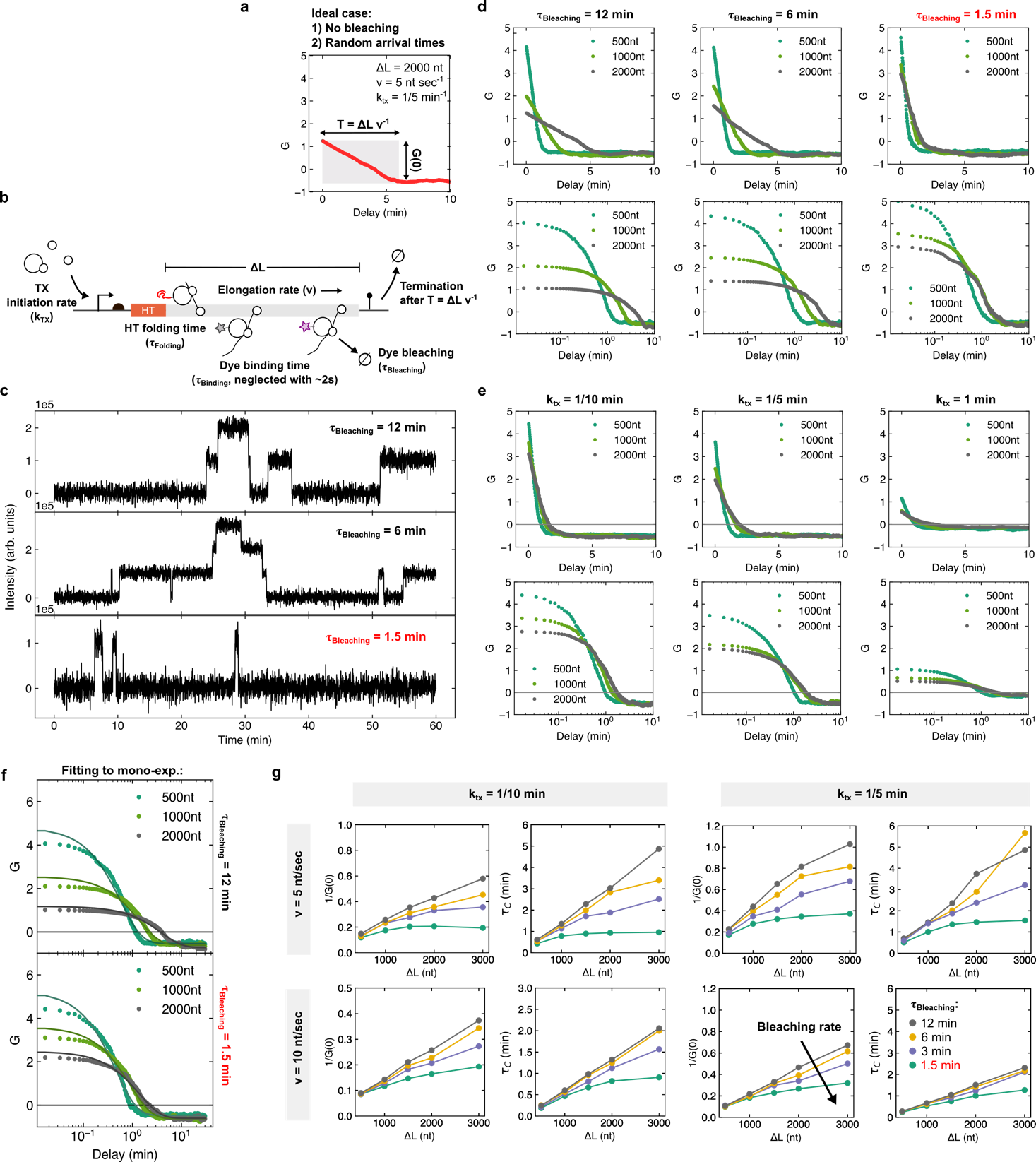


**Supplementary Figure 6: Simulations of ideal and experimentally limited ACF for proteins synthesized through coupled gene expression machines on single DNA. a)** The interpretation of the ACF for a model of nascent proteins produced randomly on a single DNA molecule. **b)** Steps in the ideal model for nascent HT proteins produced on DNA with MaP655-Halo’s fluorescent signal as read-out: Transcription initiation events with a rate *k_TX_* transitioning into transcription and translation complexes and moving along the gene with length Δ*L* and elongation rate *v*. After folding of the HT peptides (τ_Folding_ ~ 86 s) on the DNA molecule and before their release at the termination site, MaP655-Halo reacts with tethered HT proteins (τ_Binding_, neglected with ~2 s) to start fluorescing on the DNA. The fluorescent signals are either lost due to bleaching (τ_Bleaching_) or their release from the DNA after termination at time *T =* Δ*L v^-1^*. **c)** Exemplary intensity traces for simulated protein synthesis spots with different bleaching times τ_Bleaching_ and *k_TX_* = 1/5 min^-1^, *v* = 5 nt sec^-1^, and Δ*L* = 2000 nt. **d)** ACFs on a linear and log-scaled x-axis for k_tx_= 1/5 min^-1^ and indicated τ_Bleaching_ and Δ*L*. As τ_Bleaching_ becomes shorter, the ACFs transition from a linear to an exponential decay. **e)** Same as panel d for τ_Bleaching_= 1.5 min and various *k_TX_* and Δ*L*. ACF’s offset approaches zero for higher *k_TX_* and longer delays. **f)** ACF of simulated intensity traces for two τ_Bleaching_ and three Δ*L*. The mono-exponential fits to the data are shown as solid lines. **g)** The inverse amplitude, G(0)^-1^, and typical correlation time τ_C_ extracted from the mono-exponential fits as function of Δ*L* and various combinations of *v*, k_tx_, and τ_Bleaching_. We experimentally verified τ_Bleaching_= 1.5 min for MaP655-Halo bound to HT proteins (as indicated in red letters throughout this figure). Each data point is the median of 100 simulated protein synthesis spots.


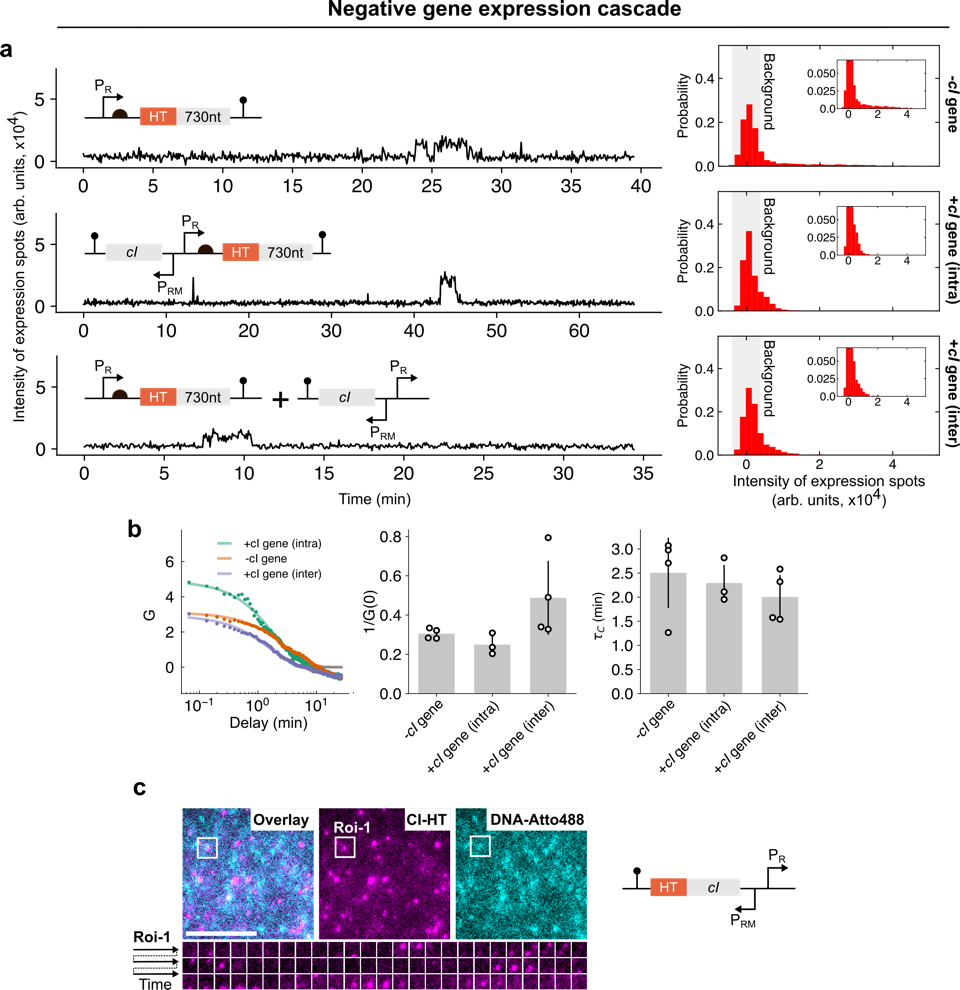


**Supplementary Figure 7: Negative cascaded gene expression reactions on the same DNA molecule. a)** Examples of protein synthesis traces (left) and intensity distributions of expression spots over the ensemble (right) for the three different experimental settings without *cI* gene (n=3 independent experiments), with *cI* gene encoded on the same DNA molecule as the *ht* gene (intramolecular case, n=3 independent experiments), and the *cI* and *ht* gene split between two DNA molecules but immobilized in the same flow channel (n=4 independent experiments). The *cI* repressor gene and regulatory elements were added to the DNA encoding the HT with 730 nt-long C-terminal extension. **b)** The ACFs with the extracted inverse amplitude G(0)^-1^ and correlation time τ_C_ for the three different setups as indicated in a. Experiments were independently replicated from left to right for n=4,3,4 (small circles) to obtain average values (bar heights) with error bars as SD. **c)** Fluorescence images of DNA and CI-HT spots (similar results with n=2 independent expression experiments). Scale bar, 5 µm. White boxes in fluorescent images show the region Roi-1 from which a time series for the signal of protein synthesis (CI-HT) was extracted (bottom montage).


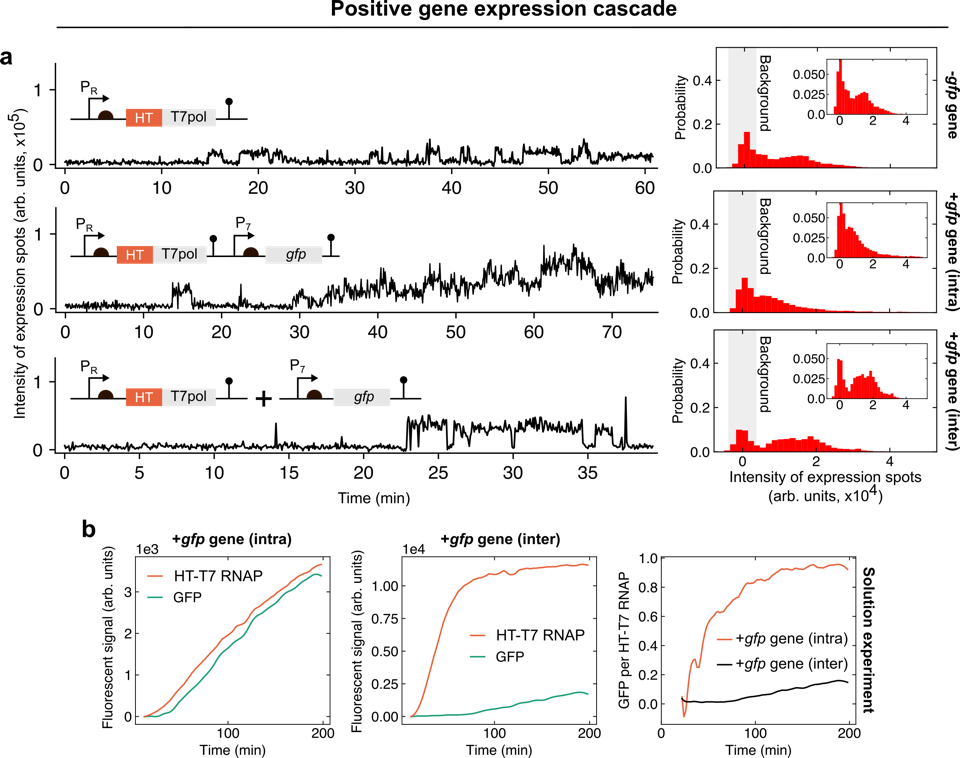


**Supplementary Figure 8: Positive cascaded gene expression reactions on the same DNA molecule. a)** Examples of protein synthesis traces (left) and intensity distributions of expression spots over the ensemble (right) for the three different experimental settings using DNA molecules encoding the gene for the T7 RNAP (T7 pol) (n=5 independent experiments), with two genes for T7 RNAP and GFP (intramolecular case, n=4 independent experiments), and genes for T7 RNAP and GFP split between two DNA molecules immobilized in the same flow channel (intermolecular case, n=3 independent experiments). **b)** Two bulk expression experiments with positive cascade encoded on a single DNA molecule (intra) and split between two different DNA molecules (inter) (each scenario with n=2 independent experiments). The rightmost panel shows the GFP signal divided by the HT-T7 RNAP signal.


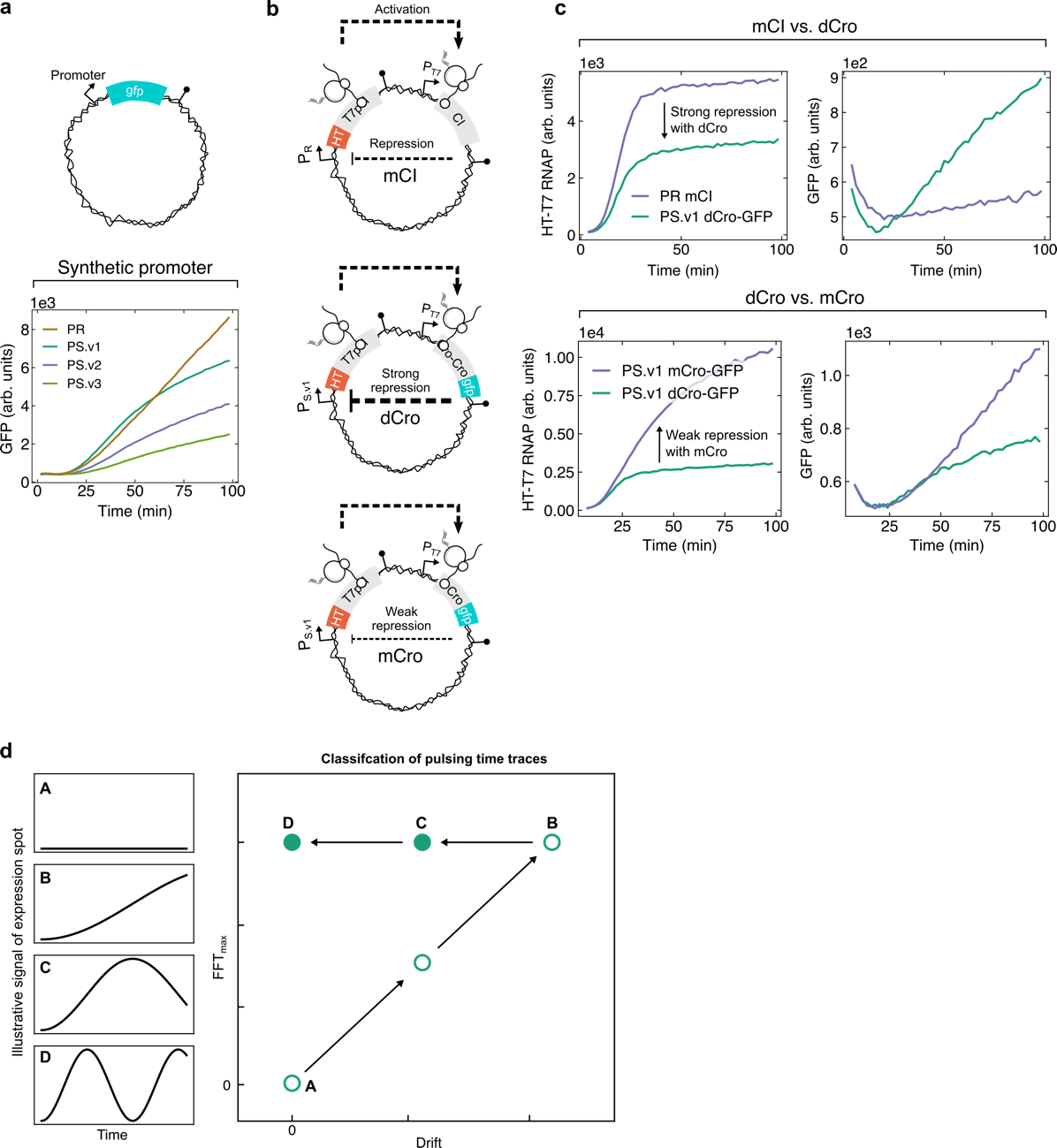


**Supplementary Figure 9: Full circuits with positive and negative feedback on the same DNA molecule. a)** Representative bulk experiments with GFP expression under control of the standard promoter P_R_ and three *de novo* designed promoters (Supplementary Table 1). The ribosomal binding sites were identical for all the constructs (n=2 experiments with similar results). **b)** Three different constructs encoding genes for the T7 RNAP and monomeric repressor cI (mCI, upper schematic), strong repressor dCro-GFP (dCro, center schematic), and weak monomeric repressor mCro-GFP (mCro, lower schematic). Each promoter controlling the transcription of HT-T7 RNAP contained two strong binding sites for the corresponding repressors (see Supplementary Table 1). **c)** First two plots show representative bulk expression experiments from the mCI and dCro constructs with HT-T7 RNAP (left panel) and dCro-GFP (right panel) signals. The auto-fluorescence of cell lysate produced a minor background signal in the GFP channel for the mCI construct (similar results for each construct with n=2 independent experiment). The last two plots show representative bulk expressions from dCro and mCro constructs with the signals for HT-T7 RNAP and dCro-GFP or mCro-GFP (similar results for each construct with n=2 independent experiments). **d)** Illustrative HT-T7 RNAP expression traces with four qualitatively different characteristics (from top to bottom): no activity, accumulation, slow pulse, two pulses. Trace A and B are located along the diagonal on a plot for drift and maximal FFT amplitude ($\mathcal{F}_{max}$). Pulsing is characterized by a constant $\mathcal{F}_{max}$, but reduced drift value (Trace C and D).

**Supplementary Table 1: DNA sequences and ribosomal binding site.**

| Name | Sequence (RBS is highlighted with capital letters) |
| --- | --- |
| N-terminal HA tag for HT (HA tag is underlined) until start codon “atg” | gcAATAATTTTGTTTAACTTTAAGAAGGAGATATAccatgaccagctacccatacgatgttccagattacgctggccgcttaattaaacatatgaccatg… |
| P_S.V1_ (Cro binding sites are underlined) until start codon “atg” | gttccgctgggcattctatcaccgcgggtgataaacttgacaactatcccttgcggtgatattatggcctgctatgcagctagcAATAATTTTGTTTAACTTTAAGAAGGAGATATAccatg… |
| P_S.V2_ (Cro binding sites are underlined) until start codon “atg” | gttccgctgggcattctatcaccgcgggtgataaaattgacatttatcccttgcggtgatataatgctatgctatgcagctagcAATAATTTTGTTTAACTTTAAGAAGGAGATATAccatg… |
| P_S.V3_ (strong CI, but weak Cro binding sites are underlined) until start codon “atg” | gttccgctgggcattaacaccgtgcgtgttgtattgacactacctctggcggtgatattattatctgctatgcagctagcAATAATTTTGTTTAACTTTAAGAAGGAGATATAccatg… |
