# Supplementary material for: A genetic circuit on a single DNA molecule as an autonomous dissipative nanodevice
Source: Nat Commun. 2024 Jan 29;15:883. doi: 10.1038/s41467-024-45186-2 (PMC10825189; doi:10.1038/s41467-024-45186-2)
Supplement: Supplementary file 3 — Description of Additional Supplementary Files [file 41467_2024_45186_MOESM3_ESM.pdf]

Description of Additional Supplementary Files Document

Supplementary Movie 1: Cell-free protein synthesis from two DNA molecules encoding the full pulsatile circuit. Two HT-T7 RNAP protein synthesis spots on DNA molecules encoding the full circuit with strong dCro-GFP repressor. The DNA (gray) slowly bleached over the course of the experiment. Upper left DNA molecule exhibited pulsatile HT-T7 RNAP (magenta) dynamics, the other nearby DNA molecule (lower right) showed accumulation of HT-T7 RNAP.

Supplementary Movie 2: Cell-free protein synthesis from five DNA molecules encoding the genetic circuit with weak mCro-GFP repressor. Five HT-T7 RNAP protein synthesis spots on DNA molecules encoding the full circuit with weak mCro-GFP repressor. The DNA (gray) slowly bleached over the course of the experiment.

Supplementary Data 1: DNA sequences of constructs created and used in this study.
