# Supplementary material for: A genetic circuit on a single DNA molecule as an autonomous dissipative nanodevice
Source: Nat Commun. 2024 Jan 29;15:883. doi: 10.1038/s41467-024-45186-2 (PMC10825189; doi:10.1038/s41467-024-45186-2)
Supplement: Supplementary file 7 — Reporting Summary [file 41467_2024_45186_MOESM7_ESM.pdf]

nature portfolio

Corresponding author(s): Ferdinand Greiss
Roy Bar-Ziv

Last updated by author(s): Dec 14, 2023

Reporting Summary

Nature Portfolio wishes to improve the reproducibility of the work that we publish. This form provides structure for consistency and transparency in reporting. For further information on Nature Portfolio policies, see our Editorial Policies and the Editorial Policy Checklist.

Statistics

For all statistical analyses, confirm that the following items are present in the figure legend, table legend, main text, or Methods section.

n/a	Confirmed	
☐	☒	The exact sample size (*n*) for each experimental group/condition, given as a discrete number and unit of measurement
☐	☒	A statement on whether measurements were taken from distinct samples or whether the same sample was measured repeatedly
☐	☒	The statistical test(s) used AND whether they are one- or two-sided<br>*Only common tests should be described solely by name; describe more complex techniques in the Methods section.*
☒	☐	A description of all covariates tested
☒	☐	A description of any assumptions or corrections, such as tests of normality and adjustment for multiple comparisons
☐	☒	A full description of the statistical parameters including central tendency (e.g. means) or other basic estimates (e.g. regression coefficient) AND variation (e.g. standard deviation) or associated estimates of uncertainty (e.g. confidence intervals)
☐	☒	For null hypothesis testing, the test statistic (e.g. *F*, *t*, *r*) with confidence intervals, effect sizes, degrees of freedom and *P* value noted<br>*Give P values as exact values whenever suitable.*
☒	☐	For Bayesian analysis, information on the choice of priors and Markov chain Monte Carlo settings
☒	☐	For hierarchical and complex designs, identification of the appropriate level for tests and full reporting of outcomes
☒	☐	Estimates of effect sizes (e.g. Cohen's *d*, Pearson's *r*), indicating how they were calculated

Our web collection on statistics for biologists contains articles on many of the points above.

Software and code

Policy information about availability of computer code

Data collection	We use Micro-Manager 1.4 and python v3.7 (with PyQt5) for the image acquisition on our single-molecule microscope. We use an Arduino control software (on Github, Nicovich et al., PloS One 12, 1-15 (2017)) for laser triggering. We used ClarioStar Plus (BMG Labtech) for analyzing solution-based expression experiments.
Data analysis	We used an adapted version of Picasso (on Github, Schnitzbauer et al., Nat. Protoc. 12 (2017)) for detecting and localizing single molecules, the Fiji distribution of ImageJ 1.51p for manual image processing, and Python v3.7 (with Matplotlib v3.3, NumPy v1.20.3, Scipy v1.3, scikit-image v0.15, numba v0.53.1) for data analysis. Custom code for data analysis is available at https://github.com/FerdinandGreiss/nanokit and a citable version at https://zenodo.org/doi/10.5281/zenodo.10370923.

For manuscripts utilizing custom algorithms or software that are central to the research but not yet described in published literature, software must be made available to editors and reviewers. We strongly encourage code deposition in a community repository (e.g. GitHub). See the Nature Portfolio guidelines for submitting code & software for further information.

Data

Policy information about availability of data

All manuscripts must include a data availability statement. This statement should provide the following information, where applicable:

- Accession codes, unique identifiers, or web links for publicly available datasets
- A description of any restrictions on data availability
- For clinical datasets or third party data, please ensure that the statement adheres to our policy

The microscopy data generated in this study have been deposited in the Zenodo database under accession code 8359540 (https://doi.org/10.5281/zenodo.8359540). Other relevant data are available within the paper as Source Data and Supplementary Information.

Research involving human participants, their data, or biological material

Policy information about studies with human participants or human data. See also policy information about sex, gender (identity/presentation), and sexual orientation and race, ethnicity and racism.

Reporting on sex and gender	N/A
Reporting on race, ethnicity, or other socially relevant groupings	N/A
Population characteristics	N/A
Recruitment	N/A
Ethics oversight	N/A

Note that full information on the approval of the study protocol must also be provided in the manuscript.

Field-specific reporting

Please select the one below that is the best fit for your research. If you are not sure, read the appropriate sections before making your selection.

☒ Life sciences ☐ Behavioural & social sciences ☐ Ecological, evolutionary & environmental sciences

For a reference copy of the document with all sections, see nature.com/documents/nr-reporting-summary-flat.pdf

Life sciences study design

All studies must disclose on these points even when the disclosure is negative.

Sample size	No statistical methods were used to calculate the sample size. The single-molecule experiments were repeated more than three times with different microfluidic chips and cell lysate on different days. When data was along a titration curve, some experiments were repeated twice with supported by the neighboring data points. The bulk solution experiments were repeated at least twice on independent days with 3 technical replicates in the same well plate. The experimental distributions for all experiments converged and the number of replicates is on par with standard biophysical experiments in the field.
Data exclusions	No data was excluded from the analysis.
Replication	We replicated experiments with different microfluidic chips (same layout), with different DNA constructs, different days, and different batches of cell lysate. The single-molecule experiments were done in randomized flow channels on the microfluidic chip (5 flow channels in total). The solution experiments were replicated with 3 technical replicates in a single well plate and again repeated (biological replicate) on other days for at least 2 times.
Randomization	No group allocation was performed.
Blinding	Blinding was not applied in this study because no group allocation was performed.

Reporting for specific materials, systems and methods

We require information from authors about some types of materials, experimental systems and methods used in many studies. Here, indicate whether each material, system or method listed is relevant to your study. If you are not sure if a list item applies to your research, read the appropriate section before selecting a response.

Materials & experimental systems

n/a	Involved in the study
☐	☒ Antibodies
☒	☐ Eukaryotic cell lines
☒	☐ Palaeontology and archaeology
☒	☐ Animals and other organisms
☒	☐ Clinical data
☒	☐ Dual use research of concern
☒	☐ Plants

Methods

n/a	Involved in the study
☒	☐ ChIP-seq
☒	☐ Flow cytometry
☒	☐ MRI-based neuroimaging

Antibodies

Antibodies used

Biotinylated high-affinity (3F10) anti-HA antibodies (50µg ml^–1, ~500nM, Sigma-Aldrich)

Validation

Standard antibody used for various experiments in our laboratory. The antibody was already validated for different studies In our laboratory, such as https://doi.org/10.1126/sciadv.aaz6020 and https://doi.org/10.1038/s41565-020-0720-7.

Plants

Seed stocks

N/A

Novel plant genotypes

N/A

Authentication

N/A
